# College Community–Based Physical Activity Support at a Public University During the COVID-19 Pandemic: Retrospective Longitudinal Analysis of Intra- Versus Interpersonal Components for Uptake and Outcome Association

**DOI:** 10.2196/51707

**Published:** 2025-06-16

**Authors:** Garrett I Ash, Selene S Mak, Adrian D Haughton, Madilyn Augustine, Phillip O Bodurtha, Robert S Axtell, Beatrice Borsari, Jason J Liu, Shaoke Lou, Xin Xin, Lisa M Fucito, Sangchoon Jeon, Matthew Stults-Kolehmainen, Mark B Gerstein

**Affiliations:** 1 Program in Computational Biology & Bioinformatics Yale University New Haven, CT United States; 2 Department of Molecular Biophysics and Biochemistry Yale University New Haven, CT United States; 3 Center for Pain, Research, Informatics, Medical Comorbidities and Education Center (PRIME) VA Connecticut Healthcare System West Haven, CT United States; 4 Center for the Study of Healthcare Innovation, Implementation & Policy (CSHIIP) VA Greater Los Angeles Healthcare system Los Angeles, CA United States; 5 Department of Health and Movement Sciences Programs Southern Connecticut State University New Haven, CT United States; 6 Department of Psychiatry Yale School of Medicine New Haven, CT United States; 7 Center for Outcomes Research and Evaluation Yale New Haven Hospital New Haven, CT United States; 8 Section of Cardiovascular Medicine Department of Internal Medicine Yale School of Medicine New Haven, CT United States; 9 Yale Cancer Center New Haven, CT United States; 10 Smilow Cancer Hospital Yale New Haven Hospital New Haven, CT United States; 11 Yale School of Nursing Orange, CT United States; 12 Center for Weight Management Yale New Haven Hospital New Haven, CT United States; 13 Department of Biobehavioral Sciences Teachers College Columbia University New York, NY United States; 14 Department of Computer Science Yale University New Haven, CT United States; 15 Department of Statistics and Data Science Yale University New Haven, CT United States; 16 Section of Biomedical Informatics and Data Science Yale University New Haven, CT United States

**Keywords:** universities, students, faculty, mobile apps, goals, social networks, education

## Abstract

**Background:**

College students are vulnerable to setting long-term trajectories of low physical activity (PA) but are reachable via mobile health fitness tracking (eg, mobile health step counting) and interpersonal support tailored to the college community. However, no studies have statistically isolated the appeal and influence of these intra- and interpersonal components in college-based PA interventions.

**Objective:**

This study retrospectively examined a college-based PA promotion program at a northeast US public university during the COVID-19 pandemic to (1) test the impact of student status on the use of intervention components and (2) determine whether such use was associated with successful retention and goal achievement in the program.

**Methods:**

The university used a commercial platform for a 30-day PA promotion program during April 2021 with intrapersonal (step-tracker syncing, education, self-monitoring, and motivational messaging) and interpersonal (friend interactions and team games) components. App use was operationalized as intrapersonal (frequency of opening app, education, and self-monitoring) and interpersonal (friends made in-app and team affiliation and size).

**Results:**

Campus-wide emails elicited sign-up by 156 undergraduate students, 57 graduate students, and 126 faculty and staff members. Objective 1 yielded the following results: undergraduates used the app less frequently (median 0.8, IQR 0.4-1.7 times per day) than other groups (graduate students: median 1.4, IQR 0.7-2.7 times per day; *P*=.01; faculty: median 1.3, IQR 0.7-2.7 times per day, H_2_=14.5; *P*=.001) but made the same number of friends (median 1-2) and teammates (median 8-9; *P*=.77 for friends and *P*=.93 for teammates). Objective 2 yielded the following results: most participants (313/335, 93.4%; 95% CI 90%-96%) were retained for the first 7 days, but by 30 days, retention dropped, most notably for undergraduate students (82/154, 53.2%; 95% CI 45%-61%), followed by graduate students (39/56, 70%; 95% CI 56%-81%) and faculty and staff (93/125, 74.4%; 95% CI 66%-82%; χ^2^_2_=12.6; *P*<.001). Retention was associated with app engagement frequency (model hazard ratio 0.56, 95% CI 0.43-0.72; *P*<.001) and affiliation with a team having high median app engagement and a large size (intracluster correlation coefficient 0.064, 95% CI 0.001-0.164, *P*=.05). Meeting a daily step goal was associated with app engagement frequency (β=.72, SE=0.21; *P*=.001), number of friends (β=.40, SE 0.20; *P*=.04), and an initial motive of maintaining or increasing (rather than starting) PA (β=.99, SE=0.21; *P*<.001).

**Conclusions:**

College students, compared with faculty and staff, used the app less frequently, used the app for a shorter duration before abandonment, and met the step goal on fewer days. Engagement with the program was associated with longer retention and better PA outcomes, which were critically modified by the interpersonal engagement. These findings suggest that college students using virtual PA support during times of physical isolation could benefit from more tailored implementation strategies (eg, timed prompts and team reassignments).

## Introduction

### Background

Physical activity (PA) levels are important determinants of long-term health outcomes. For instance, 6000 to 8000 steps per day is associated with a lower risk of health problems such as all-cause mortality, diabetes, and mental health concerns [1,2]. One critical life stage for PA support is during the college years for those who attend. For some people, college is a transition when great positive or negative change from adolescence is possible, and the lifestyle at the end of this transition is a predictor of the long-term health trajectory [3]. Studies have found that around half of college students have low PA levels [4], which further declined by 32% to 366% during the COVID-19 pandemic [5]. Thus, college is a critical window for interventions that maintain or increase PA to have a powerful and sustained impact on an individual’s life.

College students are a prime population to reach through mobile health (mHealth; ie, medicine involving mobile devices) because most consider the internet a source of health information [6,7], almost all own a smartphone, and around half use a fitness tracker or other wearable device [8]. mHealth could be a viable strategy to help college students increase their PA because their use of wearable fitness trackers and mobile apps has been associated with higher PA levels.

This association in these studies was mediated by the social aspects of using such technology (eg, real-time sharing of step counts), making it critical to understand the social context in which students are functioning. Starting college presents students with a new set of social circumstances [9]. When they leave the outside world and enter the college community, often one with cultural and geographic differences from where they grew up, there is a desire to belong and feel accepted in this new community [9]. Such an innate want for interpersonal connections is a fundamental determinant of human cognition and health outcomes including PA [10-12]. Fortunately, this new community presents unique opportunities to make social connections. First, students are assigned class instructors with high education levels and experience mentoring young adults, a cross-demographic connection less likely to occur in the outside world [9,11]. Second, students share experiences and concerns with other students such as accessing unique or licensed educational content, feeling eustress or distress about academic performance, and concerns about loneliness because of the restricted size of the college community [9,11]. On the other hand, students often struggle to initially adapt to these aspects of college [9,12]. The COVID-19 pandemic further heightened students’ unmet needs and desire for social connection [13,14]. However, when students are able to use the college environment opportunities to join peers in pursuing activities and exploring interests, these connections correlate with increased PA participation and engagement [11]. In fact, social support from friends effectively motivates PA among college students, more so than family support [15]. Yet, despite these rewards of connecting to peers of like age and education level in college, it can also be overwhelming. For instance, while social support in college correlates with situational interest in PA,1 study found that the college students’ environment was a barrier to PA because of feeling overwhelmed by the change in their first year of attendance [16]. For these reasons, college PA support must be considered and tailored to the college environment.

There are several frameworks for understanding determinants of human goal setting and accomplishment such as increasing PA. Goal-setting theory and the theory of planned behavior focus on intrapersonal determinants such as the specificity of the goal and beliefs about results from the goal. The self-determination theory also includes such intrapersonal competence but then extends to the inclusion of interpersonal relationships (ie, autonomy and relatedness) [17]. The social cognitive theory (SCT) extends beyond one-to-one and small-group interpersonal relationships to the broader sociocultural environment [18]. The SCT’s intrapersonal determinants include self-efficacy (belief in oneself to perform a behavior) and outcome expectations (the expectation that a desired outcome will result from the behavior). Its interpersonal determinants include sociocultural factors (the influences from an individual’s social and physical environment). Importantly, there is a reciprocal determinism (ie, feedback loops) between the intra and interpersonal determinants. For example, social persuasion and support from peers can boost a person’s self-efficacy to become a successful athlete. Similarly, when peers see that heightened self-efficacy and athleticism, they are even more inclined to support that person. Therefore, it is critical to study both the intra and interpersonal aspects of PA support programs.

Wearables can support the aforementioned SCT constructs. They can support self-efficacy by conveyance of personal accomplishment (eg, tracking step counts in relation to previous days or a preset goal) and verbal persuasion (eg, encouragement messages to reach a goal), as well as by offering the opportunity to leverage this self-efficacy for goal setting. Self-efficacy has been shown to impact both engagement with wearable technology [19] and behavioral outcomes resulting from its use [20]. Wearables can also support outcome expectations because the metrics they collect and display such as step counts are associated with health benefits [1,2]. Finally, wearables can support sociocultural influences through methods such as group step challenges and social media postings of achievements [21,22]. Overall, this robust applicability to SCT shows that wearables should be explored to deliver both inter and intrapersonal intervention components among college students.

Health-related interventions often have low uptake among college students [23-25]. Some reasons are related to intrapersonal aspects such as students prioritizing more pressing needs and disbelief the information was valid and applicable to them. Other reasons related to interpersonal aspects such as lack of peer involvement in designing the intervention, and the interpersonal interactions occurring over a social media platform they were not accustomed to using. Students also noted the criticality of being able to conveniently select which intervention components to use. Thus, both the intra- and interpersonal components clearly represent areas for improvement. Some preliminary evidence suggests that social support could be incorporated into mHealth PA interventions for the college student population to promote PA adherence. Findings from a systematic review of 19 papers suggest a positive association between PA and social support for PA among college students [26]. A total of 14 primary studies leveraged social interactions in college community populations to promote PA through mobile apps, including social components [21-23,27-38]. Some of these mobile apps provided an “open forum” feature (eg, Facebook-style news feed and WeChat group chat) where participants could share PA performance, interact, and provide social (ie, interpersonal) support [21,23,27-32,35,36]. Some discussed an additional feature in the mobile app which allowed participants to form ongoing “team” relationships. These relationships included an ongoing comparison of step counts with other team members (ie, a social norm) [22,27-30,38], discussions fostering mutual support (eg, complimenting success and empathizing with barriers) [21,22,33-37], and discussing educational materials on PA [22]. These activities were rewarded with team success scores, thus leveraging the established gamification strategies of cooperation [39] and socially oriented persuasion [40]. These studies incorporated a mobile app with these interpersonal components, activity trackers, and standard solitarily (ie, intrapersonally) used behavior change techniques such as text message reminders to be active, health education, goal setting, self-monitoring, and extrinsic incentives (eg, prizes). The utility for tracking devices without support from a multimodal app tailored for college students has yielded mixed results (reviewed in the study by Sultoni et al [41]), thus, multimodal communication is considered best practice for mHealth interventions [42].

One challenge with current research is determining the use and effectiveness of each of the multiple intervention components; this information is crucial to the refinement and success of the intervention [43]. Edney et al [30] found self-monitoring and ongoing team relationships were rated highly by 50% to 70% of participants compared with individual incentives and open forum social interactions, which only 20% to 30% of participants rated highly. Tong et al [29] found the self-monitoring and ongoing team relationship features were used an average of approximately 2 times per week, while open forum use was less than weekly. When the app was subsequently refined to include more personalization of the individual self-monitoring, by tailoring it to participants’ weekly progress and barriers, this approach was reported to be well-received [32]. Zhang et al [37] found participants randomly assigned to participate in an interpersonal team did not achieve greater PA increases than those randomly assigned to receive intrapersonal text messages. There is a need to build upon these mixed findings from clinical trials by statistically isolating which of the intrapersonal (ie, education, self-monitoring, and personalized advice) and interpersonal components (ie, open forum and team feature) are associated with successful engagement and PA promotion among college students in real-world settings.

### Objectives

This study aimed to build upon prior findings from clinical trials by retrospective observation of an mHealth PA promotion program incorporating both intrapersonal and interpersonal components, which was delivered with campus-wide outreach to students and faculty and staff. The first research question was how the intervention components were used and whether this use was different between students and faculty and staff, and the second question was whether such use was associated with successful retention and goal achievement in the program. We hypothesized that undergraduate students would have lower use of intervention components than graduate students and faculty and staff and that such use would be associated with successful retention and goal achievement in the program. This statistical isolation of specific components builds upon prior studies by providing insight into specific facilitators and barriers of the program, which could inform future refinement of interventions aimed at increasing PA in situations of physical isolation from peers and PA spaces.

## Methods

### Intervention Design

The American College of Sports Medicine Exercise is Medicine On Campus initiative recognizes approximately 150 higher educational institutions per year for campus PA initiatives. One of their gold-level programs, the Southern Connecticut State University (SCSU) MoveSpring Steps Challenge, was suited to our study purpose because it used mHealth delivery with backend data capture. The university initiated the challenge in response to COVID-19 pandemic policies that restricted use of their fitness and recreational facilities. It operated from April 1 to 30, 2021, and was directed by the campus fitness center, incorporating input from the campus exercise physiology club and health center. It was digitally supported by the MoveSpring mobile app platform. This platform provides customizable fitness tracking and interaction features for individual companies and organizations. SCSU and MoveSpring collaborated to program the targeted intervention with both intra- and interpersonal components (Table 1). For the passive monitoring component, users could synchronize their own device such as a fitness watch or smartphone pedometer.

**Table 1:** Program components and content.

Type and component			Content
**Intrapersonal**			
	Educational content on demand^a^	Videos Articles Actionable health tips Motivational quotes	
	Motivational content as push notifications	Topic and mini challenge of the week (eg, benefits of nature walking or post a picture of your nature walk): 2-3 times per week Campus events (eg, group walk): once per month Prize reminders: 3 times per week Achievement acknowledgment: once per week	
	Active monitoring (self-reported yes or no response)^b^	Ate ≥3-4 servings of fruits and vegetables Slept ≥7 hours Drank ≥8 glasses of water	
	Passive monitoring (device)	Steps (15,000 limit) in relation to both the personal goal^c^ and tiered cutoffs for prize raffles^d^ Minutes of moderate to vigorous physical activity^e^ Distance	
**Interpersonal**			
	Community engagement and peer support	Peer leaders and fitness center director Chat box^f^ Friend activity (average daily steps of friends) Team-specific challenges^g^ Team leaderboard (top 4 teams’ avatar, name, and average daily steps per member)	

^a^Content from MoveSpring proprietary collection and the Southern Connecticut State University student leaders related to physical activity, diet, sleep, hydration, and mindfulness.

^b^Completing ≥67% (20/30) of days ensured entry into a US $50 gift card raffle.

^c^Personal goal options were 6500, 10,000, or 12,500 steps per day.

^d^Average values of 7000, 10,000, or 12,500 steps per day ensured entry into a raffle for US $50, US $75, or US $100, respectively. One entry was awarded for days 1 to 15 and 2 entries were awarded for days 16 to 30.

^e^Meeting ≥20 min on 67% (20/30) of days ensured entry into a US $50 gift card raffle.

^f^Chat topics were wide ranging and sometimes unrelated to physical activity. This feature was only used by a few individuals, whereas the features in the subsequent 3 bullets were used by a majority of the 335 participants.

^g^The top 3 teams by average step count per member received T-shirts, grouped by the academic status of users who founded the team. Participants could actively form their own teams. Those not doing so were randomly assigned to a team in the first half of the first week of the challenge.

### Recruitment and Enrollment

Nine days before the start of the challenge (March 22, 2021), the campus fitness center coordinator sent a single advertisement email to all SCSU students and employees with instructions guiding those interested to download the SCSU-customized MoveSpring SCSU app to their personal smartphone, create an account, and synchronize a watch or pedometer for passive monitoring. Upon registration, all participants were required to complete an intake survey of academic status, campus visit frequency, reason for enrolling, daily step goal, and device used for activity tracking (Multimedia Appendix 1). To minimize the onboarding burden, there was no query on sex or other demographics.

### Data Capture

The SCSU Fitness Center and MoveSpring removed identifiers and donated the dataset to the Dataverse repository [44] and a research team based at Yale University (GIA, SSM, MA, BB, JL, SL, XX, SJ, LMF, MS-K, and MBG), who performed data cleaning, analysis, interpretation, and write-up. They liaised with several co-designers of the Steps Challenge program content (ADH, PB, and RSA) to summarize the intervention methodology for this manuscript.

### Ethical Considerations

No researchers had contact with participants or access to identifiable information, so the Yale University Institutional Review Board determined that the project did not constitute human participants research, meaning the institution waived ethical approval (determination 2000033852). This manuscript follows STROBE (Strengthening the Reporting of Observational Studies in Epidemiology) guidelines for cohort studies (Multimedia Appendix 2). Participant identifiers were removed before the transfer of the data to Dataverse and Yale.

### Data Analysis

#### Overview

This study was a retrospective analysis of longitudinal observations on campus community members over the 30 days they were exposed to a steps challenge intervention app. Analyses used SPSS (version 26.0; IBM Corp; aim 1) and SAS (version 9.4; aim 2). Significance was set at .05. Study size was based on the available dataset.

#### Outcomes

The first objective was to determine how the app was used and whether app use differed by academic status. The outcomes for this first objective were app use metrics, operationalized as intrapersonal (average frequency of opening the app, education, and self-monitoring as continuous variables) and interpersonal features (friends made in-app, team affiliation, and team size as discrete variables). The second objective was to determine whether app use was associated with successful retention and goal achievement in the program. The outcomes for this second objective were (1) days of retention quantified as a time-to-event discrete variable censored at the end of the 30-day exposure (ie, the event being the discontinuation of app syncing) and (2) each day’s success at meeting the step goal, quantified as a ternary categorical variable (tracking device not worn, tracking device worn but not reaching goal, tracking device worn, and reaching goal). The first objective analyzed outcomes at the person level (ie, daily averages), while the second objective analyzed outcomes at the day level adjusted for within-person correlations. These units were chosen based on the available granularity of the data from the app that both delivered the intervention and tracked its outcomes.

#### Research Question 1: How Was the App Used, and Did App Use Differ by Academic Status?

Participant characteristics (Table 2) were tabulated as frequencies and compared by academic status using chi-square tests (χ^2^). The duration of app use before abandonment (Table 3) was compared by academic status using the Kaplan-Meier estimate of survival probability because it was right-censored at the 30-day length of the program. Most metrics of app use before abandonment (Table 3) were compared by academic status using Kruskal-Wallace (H) tests as they were interval scale variables with distributions that were bimodal (number of teammates) or right skewed (frequency of opening the app before abandonment, friends made in-app, and use of the self-monitoring and education features) and visually similar between the academic statuses. Two metrics of app use before abandonment were even more heavily right skewed—use of the self-monitoring and education features—so they were recoded as binary (<1 time weekly or ≥1 time weekly) and compared by academic status using chi-square tests. All variables with a significant main effect across the 3 academic groups were further tested by post hoc pairwise comparisons with *P* value multiplied by 3 to Bonferroni-adjust for multiple comparisons.

**Table 2:** Participant descriptive characteristics.

			Overall		Undergraduates		Graduates		Faculty and staff		Chi-square (*df*)			*P* value	
Participants, n			339		156		57		126		N/A^a^			N/A	
**Campus visit frequency, n (%)**															
	Remote	82 (24)		34 (22)		32 (56)		16 (13)		71.9 (4)			*<.*001^b^		
	Regularly visit	102 (30)		61 (39)		18 (32)		23 (18)							
	Live or work	155 (46)		61 (39)		7 (12)		87 (69)							
**Step count mechanism, n (%)**												—^c^			—
	Apple	207 (61)		110 (71)		41 (72)		56 (44)							
	Fitbit	63 (19)		22 (14)		12 (21)		29 (23)							
	Garmin	14 (4)		4 (3)		1 (2)		9 (7)							
	Withings	1 (0.3)		0 (0)		0 (0)		14 (11)							
	Google	25 (7)		11 (7)		0 (0)		1 (1)							
	Manual entry	21 (6)		5 (3)		2 (4)		14 (11)							
	Dropped out before syncing	8 (2)		4 (3)		1 (2)		3 (2)							
**Motive for joining the program, n (%)**												2.5 (4)			.65
	Start physical activity	68 (20)		31 (20)		10 (18)		27 (21)							
	Increase physical activity	102 (30)		44 (28)		22 (39)		36 (29)							
	Maintain physical activity	169 (50)		81 (52)		25 (44)		63 (50)							
**Step goal, n (%)**												0.8 (4)			.94
	6500	176 (52)		82 (53)		30 (53)		64 (51)							
	10,000	132 (39)		62 (40)		21 (37)		49 (39)							
	12,500	31 (9)		12 (8)		6 (11)		13 (10)							

^a^N/A: not applicable.

^b^Italicized values indicate *P*<.05.

^c^Not compared across groups due to small cell sizes.

**Table 3:** Use of app by academic status.

					Overall			Undergraduates			Graduates			Faculty and staff			Test statistic			*P* value
Participants, n					335			154			56			125			—^a^			—
**Overall use**																				
	**Days of app use before abandonment, n (%)**																12.6 (*df*=2)^b^			*.*002^c^
		>7	313 (92)			145 (93)			51 (90)			117 (93)								
		>14	291 (86)			126 (81)			49 (86)			116 (92)								
		>21	260 (77)			110 (71)			46 (81)			104 (83)								
		>28	226 (67)			88 (56)			42 (74)			96 (76)								
		30	214 (63)			82 (53)			39 (68)			93 (74)								
	**App openings per day before abandonment, median (IQR)**			1.1 (0.5-2.3)			0.8 (0.4-1.7)^d^			1.4 (0.7-2.7)			1.3 (0.7-2.7)			14.5 (*df*=2)^e^			*<.*001^c^	
		≥1 time weekly, n (%)	304 (91)			138 (90)			54 (96)			112 (90)								
		≥1 time daily, n (%)	190 (57)			71 (46)			39 (70)			80 (64)								
		≥2 times daily, n (%)	95 (28)			32 (21)			21 (38)			42 (34)								
	**Friends made, median (IQR)**			2 (0-3)			2 (0-3)			1 (0-4)			2 (0-3)			0.5			.77	
		≥1, n (%)	211 (63)			96 (62)			35 (63)			80 (64)								
		≥2, n (%)	168 (50)			77 (50)			27 (48)			64 (51)								
		≥3, n (%)	116 (35)			49 (32)			18 (32)			49 (39)								
		≥6, n (%)	36 (11)			14 (9)			5 (9)			17 (14)								
	**Teammates made, median (IQR)**			9 (6-10)			9 (6-10)			9 (5-10)			8 (6-10)			0.1			.93	
		1, n (%)	1 (1)			0 (0)			1 (1)			0 (0)								
		3-5, n (%)	70 (21)			31 (20)			15 (27)			24 (19)								
		6-9, n (%)	132 (39)			62 (40)			15 (27)			55 (44)								
		≥10, n (%)	132 (39)			61 (40)			25 (45)			46 (37)								
	Sleep logged^f^, n (%)			54 (16)			23 (15)			15 (27)			16 (13)			5.9 (*df*=2)^g^			.053	
	Diet logged^f^, n (%)			40 (12)			18 (12)			9 (16)			13 (10)			1.2 (*df*=2)^g^			.55	
	Hydration logged^f^, n (%)			62 (19)			28 (18)			15 (27)			19 (15)			3.5 (*df*=2)^g^			.18	
	Education viewed^f^, n (%)			9 (3)			4 (3)			3 (5)			2 (2)			2.1 (*df*=2)^g^			.35	

^a^Not applicable.

^b^Chi-square value of the Kaplan-Meier log rank.

^c^Italicized values indicate *P*<.05.

^d^Pairwise results: undergraduate was lower than graduate (H_1_=43.6, adjusted *P*=.01) and faculty (H_1_=35.9, adjusted *P*=.006). Graduate and faculty were not different (H_1_=0.5, adjusted *P*>.99).

^e^H value of the Kruskal-Wallace test.

^f^Recoded as binary (<1 time weekly or ≥1 time weekly) due to substantial right-skew.

^g^Chi-square value of binary comparison.

#### Research Question 2: Was App Use Associated With Successful Retention and Goal Achievement in the Program?

Independent variables were app use metrics (engagement frequency, number of friends made, and number of teammates made) and all available participant characteristics (academic status, campus visit frequency, motive for joining the program, and step count goal). Each of the following analyses tested each independent variable’s bivariate association with the retention or goal achievement outcome; those meeting a *P*<.10 cutoff were tested in a multiple variable model, and those appearing to influence that model (*P*<.20) were retained in the final parsimonious model.

**Figure 1:**
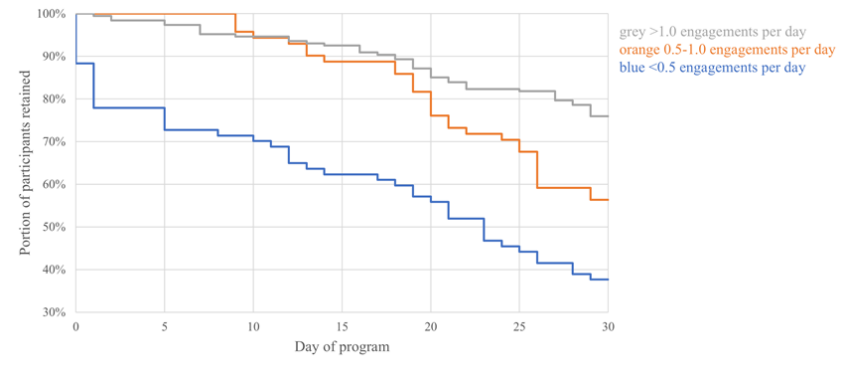
Retention survival curve according to engagements per day, which was the only predictor of retention retained in the parsimonious final model.

**Figure 2:**
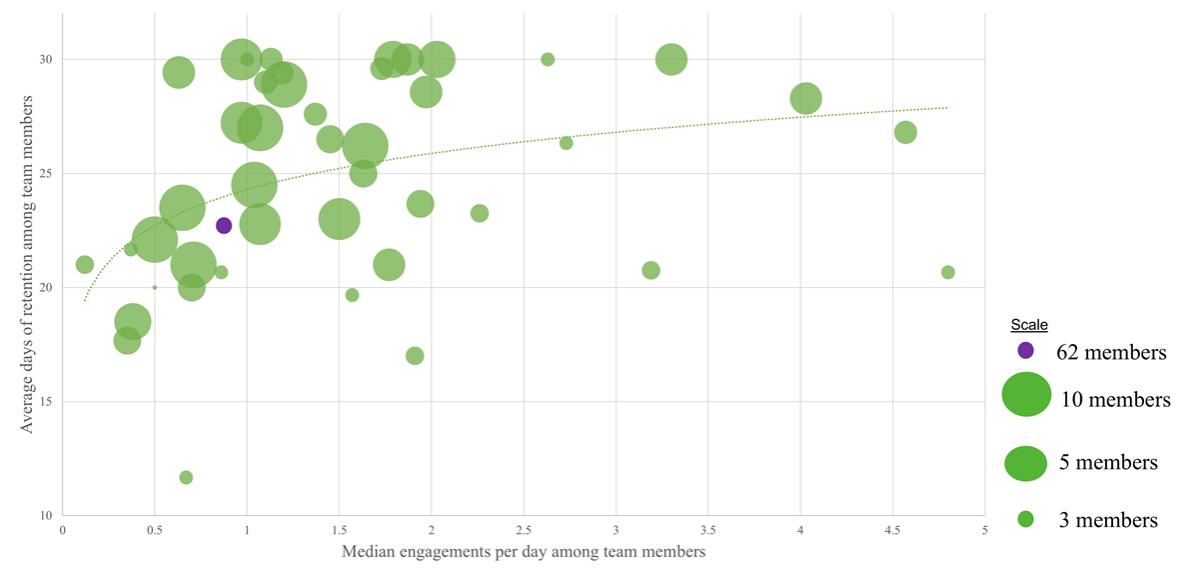
Retention (ie, duration of app use before abandonment) was clustered by team affiliation (intracluster correlation coefficient 0.064, 95% CI 0.001-0.164; *P*=.05). Therefore, we plotted average retention among team members in relation to team characteristics, including team size (bubble size) and median app engagements per day among team members (x-axis). It is visually apparent that the longer-retained individuals were those on teams with a larger size and higher median rate of app engagement. The dotted curve is for visual purposes and approximates retention days as 2.29 × ln(median engagement of team) + 24.29.

Retention (ie, duration until last time the app was opened before abandonment) was estimated using the Kaplan-Meier estimate, and effects of independent variables were examined using a Cox frailty proportional hazard model incorporating correlation within teams. Besides accounting for the right censorship of the data, these methods can adjust for possible correlations within the dataset; in this case, the correlation between teammates. The lone influential independent variable was visualized using a survival curve (Figure 1), and correlation within teammates was visualized as a bubble plot (Figure 2).

Goal achievement was labeled for each person-day as device nonwear (0 steps), wear-below-goal (steps nonzero but below goal), or goal (steps at or above goal). We tested independent variables associated with nonwear versus goal and wear-below-goal versus goal, using a generalized estimating equation that incorporated correlation within participants by a working correlation structure, a random intercept, and team affiliation as a random effect (Table 4). We also ran a supplemental analysis where the outcome variable was the total steps per day among participants meeting the standard minimum representative PA sampling of 4 days [45] (Multimedia Appendix 3).

**Table 4:** Generalized estimating equation of day-level step-goal achievement among 8287 daily observations occurring before app abandonment.

				Below goal (*k*=4659) vs at goal (*k*=3548)				Nonwear (*k*=80) vs at goal (*k*=3548)		
				Adjusted ꞵ (SE)^a,b,c^		*P* value		Adjusted ꞵ (SE)^a,b,c^		*P* value
**Baseline characteristics**										
	**Academic status**									
		Undergraduate student	.35 (0.16)		*.*03^d^		.82 (0.53)		.12	
		Graduate student	.50 (0.22)		*.*02^d^		−.21 (0.76)		.78	
		Faculty and staff (reference group)	—^e^		—		—		—	
	**Motive for joining**									
		Maintain physical activity	−.99 (0.21)		*<.*001^d^		−1.69 (0.70)		.02^a^	
		Increase physical activity	−.63 (0.22)		*.*01^d^		−1.70 (0.70)		*.*02^a^	
		Start physical activity (reference group)	—		—		—		—	
	**Goal**									
		12,500	.38 (0.27)		.15		.35 (1.05)		.74	
		10,000	.35 (0.16)		*.*03^d^		.80 (0.48)		.09	
		6500 (reference group)	—		—		—		—	
**Use of app features**										
	**App engagements per d before abandonment**									
		>1.00	−.72 (0.21)		*.*001^d^		−.81 (0.61)		.19	
		0.50 to approximately 1.00	−.36 (0.23)		.12		−.60 (0.60)		.31	
		<0.50 (reference group)	—		—		—		—	
	**Teammates made**									
		≥9	−.41 (0.27)		.13		−1.08 (0.76)		.16	
		5-8	−.37 (0.27)		.17		−1.40 (0.81)		.09	
		0-4 (reference group)	—		—		—		—	
	**Friends made**									
		≥4	−.40 (0.20)		*.*04^d^		−.69 (0.88)		.44	
		1-3	−.33 (0.18)		.07		−.24 (0.46)		.60	
		0 (reference group)	—		—		—		—	

^a^Positive coefficient indicates a greater likelihood of “below goal” or “not-wear.”

^b^Within-subject correlations were incorporated using a working correlation structure.

^c^The “team affiliation” random effect was ignorable because its estimated variance was very small compared with the variance of the random intercept.

^d^Italicized values indicate *P*<.05.

^e^Not applicable.

## Results

### Enrollment

#### Overview

For the 2020 to 2021 school year, there were 11,055 individuals affiliated with SCSU (7047/11,055, 63.74% female, 6287/11,055, 56.87% non-Hispanic White) as either an undergraduate student (7440/11,055, 67.3%), graduate student (1891/11,055, 17.11%), or faculty and staff member (1724/11,055, 15.59%; Multimedia Appendix 4) [46]. Among these individuals, all of whom received a recruitment invitation email, 339 (3.1%) respondents registered for the steps challenge. A total of 156 (2.1%) undergraduate students, 57 (3%) graduate students, and 126 (7.3%) faculty and staff members enrolled in the program relative to the total SCSU population. All 339 enrolled individuals were included in the sample description (Table 2), although 4 were excluded from the subsequent analysis owing to missing data on the MoveSpring backend.

#### Campus Visit Frequency

Most undergraduate students (122/156, 78.2%) frequently visited campus, of whom about half reported also residing on campus. Conversely, only 44% (25/57) of graduate students frequently visited campus, of whom the majority reported residing off campus. Nearly all faculty and staff (110/126, 87.3%) worked on campus but lived off campus as the university did not have faculty and staff housing.

#### Mechanism to Sync Step Count

Most participants (284/339, 83.8%) synchronized a device brand that most studies in a quantitative systematic review found to meet Consumer Technology Association validity standards (<10% mean absolute percent error; ie, Apple, Fitbit, or Garmin) [47]. Some (25/339, 7.5%) used a Google product (eg, Android phone step counter) to collect step count data, with faculty and staff members using this feature more than undergraduate and graduate students. In total, 6.2% (21/339) of participants manually entered step count, but it was unclear how the data were collected; 2.4% (8/339) of participants did not synchronize a device after sign-up and thus did not report any step count data.

#### Motives and Goals

Among possible motives for program enrollment, 20.1% (68/339) enrolled to initiate PA, 30.1% (102/339) to increase PA, and 49.8% (169/339) for PA maintenance. Among these 169 “maintainers,” step goals were as follows: 75 (44.4%) chose 6500 steps, 76 (45%) chose 10,000 steps, and 18 (10.7%) chose 12,500 steps. These proportions were similar to the 102 “increasers:” (n=55, 53.9%, 6500 steps; n=39, 38.2%, 10,000 steps; and n=8, 8%, 12,500 steps; χ^2^_2_=2.4, *P*=.30) but higher than the 68 “starters” (n=46, 68%, 6500 steps; n=17, 25%, 10,000 steps; and n=5, 7%, 12,500 steps; χ^2^_2_=10.6; *P*=.005).

### Research Question 1: Use of the App Overall and by Academic Status

#### Duration Before Abandonment

Most (313/335, 93.4%) participants stayed in the program for the first 7 days, and 63.9% (214/335) stayed for the full 30 days. At all time points beyond the first week, retention among undergraduate students tended to be lower than among graduate students and was lower than among faculty and staff (Table 3).

#### Frequency Before Abandonment

Most participants opened the app with some regular frequency—at least 0.5 times per day—but undergraduates did so less frequently than both faculty and staff and graduate students. Lower values of this metric were associated with both sooner abandonment and fewer step counts logged before abandonment.

#### Self-Monitoring and Education Features

In addition to regularly opening the app as described earlier, almost all participants (331/335, 98.8%) used at least one of the educational or self-monitoring features at least once to collect data about sleep, diet, and hydration. At the same time, the proportions using them at least weekly were much smaller: accessing education content (9/335, 2.7%), recording sleep data (54/335, 16.1%), recording diet (40/335, 11.9%), and recording hydration (62/335, 18.5%). Use of these features did not differ by academic status, although sleep logging had a nonsignificant tendency to be more common among graduate students than among undergraduates or faculty and staff.

#### Social Friends and Teams

Most participants made at least 1 friend in the app, and half made multiple friends. All participants were part of a team, which ranged in size from 2 to 62 members. Most teams comprised ≥6 individuals. Faculty and staff members were most often part of a team of 6 to 9 participants, and graduate students were most often part of a team of ≥10 participants. Most undergraduate participants were split between being in a team of 6 to 9 and a team of ≥10 participants. We did not detect statistically significant differences between the number of friends made or the number of teammates by academic status.

### Research Question 2: Association of App Use With Successful Retention and Goal Achievement in the Program

#### Variables Associated With Retention

The final model of variables associated with retention (ie, duration of use before abandonment) included app engagement frequency (Figure 1) with adjustment for correlation within teams. Overall frailty model hazard ratios for abandonment were 0.56, 95% CI 0.43-0.72; *P*<.001 for those in the first and second quartile of engagement frequency (≥1.0/d), and 0.69, 95% CI 0.50-0.96; *P*=.03) for those in the third quartile of engagement frequency (0.5-1.0/d), compared with those in the fourth quartile of engagement frequency (<0.5/d). At 10 days, abandonment was highest among those in the fourth quartile compared with the 3 other quartiles (30% vs 5%). At 30 days, abandonment was again highest among those in the fourth quartile (62%), followed by those in the third quartile (44%) and those in the first and second quartiles (24%).

The intraclass correlation coefficient by teams was 0.064 (0.001-0.164); *P*=.05. Specifically, the longer-retained individuals were those on the teams with larger size and higher median rate of app engagement (Figure 2).

#### Variables Associated With Step-Goal Achievements

Among the 10,050 participant-days, 1763 occurred after app abandonment. Of the remaining 8287 participant-days, there were 3550 where steps meeting or exceeding goal amount were recorded, 4670 where steps exceeding a reasonable daily value (>250) but not meeting goal amount were recorded, and 80 days (among 28 participants) where no steps were recorded indicating nonwear of the device. On days the device was worn, the median step count was 6756 (IQR 4649-9793).

Both failing to meet the step goal and failing to wear the watch were less likely for individuals whose initial motive was maintaining or increasing PA, compared with those whose initial motive was to start PA (Table 4). Failing to meet the step goal was also less likely for individuals who engaged most frequently with the app and made a high number of friends. Finally, failing to meet the step goal was less likely for faculty and staff compared with students. When this analysis of meeting the step goal was repeated on average step counts, the associated independent variables were the same (Multimedia Appendix 2).

#### Exploratory Analysis: Role of Gift Card Incentives as Possible Confounder

**Figure 3:**
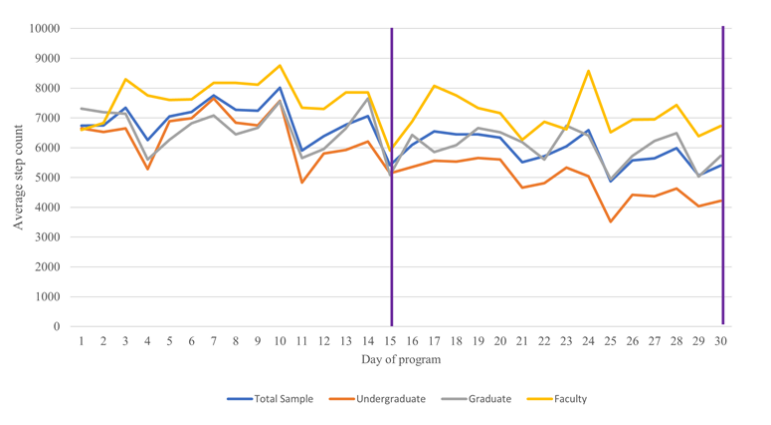
Temporal trends in steps per day relative to the cutoffs for the prize drawings (purple).

The escalation of incentive for extrinsic goals in the second half compared with the first half (ie, 2 raffle entries instead of 1) was not associated with a positive change in attaining these goals: among the 182 who did not meet the 7000 steps threshold in the first half, only 11 (6%) met it in the second half, whereas among the 157 who met the 7000 steps threshold in the first half, 36 (23%) did not meet it in the second half. A similar 4- to 10-fold smaller preponderance of upward versus downward transitioners was apparent at the step cutoffs of 10,000 (17/247, 6.9% vs 20/75, 27%) and 12,500 (8/304, 2.6% vs 8/27, 30%). In addition, visually, there was no indication that participants made a “sprint finish” as they approached day 15 or day 30 to qualify for the entries of that period (Figure 3).

The incentive of a drawing for completing self-reports on ≥67% (20/30) of days also appeared uninfluential as no participants met this goal. The incentive of a drawing for achieving 20 minutes of moderate to vigorous activity on ≥67% (20/30) of days could not be evaluated for influence because the database did not contain this metric on a day level. The incentive of receiving a T-shirt for being a top 3 team was not separable from the intrinsic motivation associated with being on a successful team.

## Discussion

### Principal Findings

We assessed intrapersonal and interpersonal factors to better understand facilitators and barriers to college community–based PA support at a northeast public university during the COVID-19 pandemic. For intrapersonal factors, most participants engaged solitarily with the app at least several times per week, and this frequency was positively associated with both retention and step-goal achievement while retained. College students, when compared with faculty and staff, (1) used the app less frequently; (2) used the app for a shorter duration before abandonment; and (3) were less successful in step count goal achievement. For interpersonal factors, students and faculty and staff equally used the open friends forum and the ongoing teams feature, which were positively associated with step-goal achievement and retention before abandonment, respectively. In fact, retention before abandonment clustered among teammates (Figure 2) with a similar intracluster correlation coefficient as various PA metrics cluster among high school classmates [48]. Overall, it is important to note this interaction between intrapersonal (ie, use of app) and interpersonal (ie, social support) characteristics.

College students are vulnerable to establishing a lifelong trajectory of low PA levels. Findings from our study suggest that expanding interventions beyond self-monitoring to a multimodal intervention including interpersonal components may help address engagement and retention. The multicomponent mHealth PA promotion program provided insight into enrollment, use of specific app features, retention, device wear, and achievement of step goals in a real-world setting of a public university during pandemic circumstances, which had presented barriers to PA. Furthermore, the sample was reflective of US 4-year public university averages with respect to the proportion of students living on campus (2.1 million/5.8 million, 37%) [49], studying remotely (2.1 million/9.1 million, 23%) [50], and choosing Apple tracking devices (567/768, 73.8%) [51]. This situation presented an opportunity to retrospectively observe the intervention in the naturalistic college community environment with individuals having varying degrees and types of participation.

### Comparison With Prior Work

Teams were included in this program because small-group team challenges within a fitness program improve individual challenge completion and the degree of participation [52]. Tong et al [29] and Edney et al [30] found that ongoing team relationships were a relatively popular aspect of such an intervention among college students, more so than open forum use. Similarly, we found that most of the social interaction on our app occurred in the “teams” rather than the “friends” feature. Our additional multiple variable and multilevel (person and team) analyses revealed that differential use of both intra- and interpersonal components critically modified outcomes in step counting and retention. This finding builds upon 2 previous studies that revealed correlations between intrapersonal component use (goal setting and content completion) and increased PA [53,54]. There is a need for further research with additional methodologies to provide further insight into how we could better deploy engagement strategies such as “teams” or “open forum friends” features to promote PA.

The SCSU-customized MoveSpring program was highly accessible to the college population because participants were able to participate from anywhere. Accessibility during this time was crucial due to COVID-19 pandemic restrictions creating a very inaccessible environment for PA. The steps challenge enabled participants to stay engaged within a social network, despite being physically isolated from their friends, family, and prior PA partners during the COVID-19 pandemic. Individuals were able to join teams with other individuals they may not have known previously. This virtual connection allowed individuals to immediately have at least one other individual on their campus also participating in the same activity.

However, because using an intensive recruitment and retention strategy would have been challenging during the pandemic, the program adopted a more pragmatic approach to implementation, which revealed a more true-to-life scenario. Naturalistic studies provide an opportunity to observe patterns in college students’ PA patterns. In addition to examining participant results from an individual point of view, including teams in the intervention provided additional information about the potential of virtual teams in facilitating program engagement and retention, even in situations where participants are separated from each other and common PA spaces (eg, rural areas, satellite campuses, web-based degree programs). As college students can be a hard-to-engage population, having this knowledge, as well as understanding their PA habits can better inform future implementation and intervention efforts.

First, intervention planners should consider the added value of web-based interaction within an educational community and its ability to withstand barriers of geography and cost. The SCSU-customized MoveSpring program was accessible to those not physically on campus, incurred zero cost of devices (everyone used their own device), and had peer uptake and interactions that were mostly automatically facilitated by the app’s team features. The minimal human facilitation required was achievable by student leaders and the fitness center director without extra staffing required in a minimal resource environment.

Second, these findings suggest that college students could benefit from a more tailored implementation strategy. For example, retention prompts could be targeted at those with the lowest overall engagement early in the program, and at those with medium engagement after 3 weeks of the program, in accord with when these respective groups fell below the highest engaged group in retention (Figure 2). Such anticipation of vulnerability to abandonment has been done elsewhere [55]. In addition, these prompts could include team reassignment to teams with high engagement to leverage the clustering of engagement by team affiliation. This strategy aligns with recent recommendations that interventions should be examined not only for their overall effects but also for the contribution of individual components to determine the active versus unnecessary ingredients and achieve tailoring to individuals and contexts [43]. Further tailoring could be attained by a machine learning prediction algorithm to trigger just-in-time adaptive intervention prompts.

### Limitations and Strengths

This study had several limitations. First, although the program was offered campus-wide to recruit a representative sample, only 3.1% of the campus participated, which could lead to biased results. However, this representation is higher than the typical clinical trial enrollment of a population where often <3% even click the advertisement and <1% can commit to the logistical burdens of participation [56]. Nonetheless, future programs could make more extensive recruitment efforts to increase representation. Second, because it was a retrospective evaluation of a real-world program rather than a human subjects research study, we did not have survey assessments to test theoretical mechanisms (eg, self-efficacy and outcome expectations to test the SCT) nor participant demographics other than identification as undergraduate, graduate, or faculty and staff. Collecting more demographic and theoretical construct data would help target key program factors and their relationship with PA outcomes, accounting for theoretical mechanisms and diversity of participants. However, in young adulthood, the stage of independence or education is a more meaningful metric than biological age [57]. Third, also to reduce the burden of sign-up, participants used their own step-counting device. Almost all were previously validated up to consumer standards, but they nonetheless varied in brand and likely in model. However, the study outcome was behavioral goal achievement rather than a surrogate of the ground truth step [2]. Fourth, the program had a relatively short duration. Although, in our study and studies conducted by Lattie et al [14] and Adu et al [58] the first month of app use was critical regarding emergent differences between individuals in retention and degree of participation, so it was an appropriate timeframe for our research question. Finally, we isolated the inter- versus intrapersonal component comparison by controlling for within-person correlations statistically and within-environment correlations by using a single site, which held a gold-level designation. Nonetheless, it could also be useful to run a between-site design in which an alternate “control” site offered fewer of the components. It might also be useful to set up a design isolating intrinsic from extrinsic (ie, prize incentives) motivation, but our brief analysis of the latter indicated a lack of influence.

While prior clinical trials evaluated interpersonal college community–based PA support [27-30,32], a unique strength of our study was addressing its real-world potential for retention, engagement, and association with step-goal achievement. Moreover, we ran this evaluation at the level of individual intervention components across the spectrum from intra to interpersonal. Doing so required detailed statistical analysis including survival curves and models including multiple variables and levels (person and team). This analysis was a critical advancement from prior studies that tested such associations only univariately and only for intrapersonal not interpersonal components [53,54]. Finally, the study was strengthened by its timing: it occurred during the COVID-19 pandemic before vaccine rollout to young adults, which was a time of extensive barriers to both PA and social interactions. Moreover, it was the month before the university’s final examinations when college mHealth interventions were vulnerable to abandonment [24]. Thus, the results will inform guidance for coping with difficult times for PA and social interactions.

### Future Directions

Clinical trials cannot address the real-world potential for retention and engagement by these applications. App disengagement and abandonment is prevalent in real-world settings according to survey data [59]. In direct assessments of interventions, while these metrics are challenging to standardize across studies, a recent systematic review found only 9% of 62 published studies considered their retention and engagement to be successful [60]. Retention was 10 times less likely if participants were not financially compensated and 4 times less likely if the app was for general lifestyle maintenance rather than for an existing clinical condition [60]. Although none of the included studies had an average age <30 years, other researchers have reported that college students are challenging to retain [23]. As such, despite the availability of pre-post outcomes on retained participants, there is a dearth of information regarding how to achieve retention and engagement, especially for preventive interventions for college students in real-world settings without financial compensation for participation. These retention issues also speak to concerns about the representativeness of the select group that is successfully recruited and retained. While concerns about representativeness apply to any research, the topic of social support approaches is particularly affected because it depends on the environment of these interactions; there is a need for open forums that represent the full university sociocultural environment rather than the subsample who met the criteria and commitment for research procedures.

We also considered that lack of goal achievement can result not only from lower-than-desired step counts but also from nonwear of the tracking device. In our analysis, these 2 occurrences were both associated with a motive for joining the program being the start rather than the increase or maintenance of PA. These findings suggest that the act of wearing and monitoring a fitness wearable has a motivational etiology, rather than simply being a methodological concern as it is usually treated in research contexts. Stricter study procedures with more controlled observation can sometimes cause participants to act differently than they would usually (ie, Hawthorne bias), potentially leading to different outcomes when implemented in a real-world setting. On the other hand, successful step-goal achievement on days the watch was worn was associated with variables that were not relevant to successful watch wear, including high engagement and a high number of friends. Thus, although having similar etiology, these 2 behaviors may require different interventions.

Finally, the intervention and analyses reported herein among several hundred participants from a single campus should be repeated at other campuses to test generalizability. Efforts should be made to engage campuses from the American College of Sports Medicine’s Exercise is Medicine On Campus initiative lists and others for means that interventions can collect data and transfer it to research teams. The MoveSpring platform would be one excellent mechanism; many institutions offer it to faculty and staff but should consider offering it to students, as in this study.

### Conclusions

Engagement with the program suggests longer retention and better PA outcomes, which were critically modified by the interpersonal aspects of this engagement. Adopting a pragmatic approach to implementation highlights opportunities for future trials and the need for specific strategies tailored to different groups to account for differences in demographics and baseline motives and goals.

## Appendix

Multimedia Appendix 1 Intake survey.

Multimedia Appendix 2 STROBE checklist.

Multimedia Appendix 3 Supplemental analysis.

Multimedia Appendix 4 Campus demographics.

## Abbreviations

mHealth: mobile health
PA: physical activity
SCSU: Southern Connecticut State University
SCT: social cognitive theory
STROBE: Strengthening the Reporting of Observational Studies in Epidemiology

## Data Availability

All data analyzed in this study are publicly available on Dataverse [44].
