# Supplementary material for: College Community–Based Physical Activity Support at a Public University During the COVID-19 Pandemic: Retrospective Longitudinal Analysis of Intra- Versus Interpersonal Components for Uptake and Outcome Association
Source: JMIR Mhealth Uhealth. 2025 Jun 16;13:e51707. doi: 10.2196/51707 (PMC12209730; doi:10.2196/51707)
Supplement: Multimedia Appendix 1 [file mhealth_v13i1e51707_app1.docx]

Appendix 1: Intake Survey

1. I am a:

- Undergraduate Student
- Graduate Student
- Faculty or Staff

1. My connection to the SCSU campus is:

- I do not visit campus
- I currently visit campus regularly
- I currently live or work on campus

1. I am participating because I want to:

- Start to become more physically active
- Increase my level of physical activity
- Continue being physically active

1. My step goal is:

- Averaging 6,500 steps (3.25 miles) or more a day
- Averaging 10,000 steps (5 miles) or more a day
- Averaging 12,500 steps (6.25 miles) or more a day

1. I count steps by:

- Apple
- Fitbit
- Garmin
- Google
- Withings
- Manual entry
