# Supplementary material for: College Community–Based Physical Activity Support at a Public University During the COVID-19 Pandemic: Retrospective Longitudinal Analysis of Intra- Versus Interpersonal Components for Uptake and Outcome Association
Source: JMIR Mhealth Uhealth. 2025 Jun 16;13:e51707. doi: 10.2196/51707 (PMC12209730; doi:10.2196/51707)
Supplement: Multimedia Appendix 3 [file mhealth_v13i1e51707_app3.docx]

# Appendix 3

## Method

To complement the analyses of step goal achievement and device wear (Table 4 from main text), we also ran a supplemental analysis of total steps per day among participants meeting the standard minimum representative PA sampling of 4 days. We estimated least squares means and standard errors of daily steps using a generalized linear mixed model incorporating correlation within teammates. When possible, missing days were imputed by averaging two variables: the person’s average steps per day during that week, and the person’s average steps per day for that day of the week.

## Results

We identified 315 / 335 participants (94%) who synced at least 4 days of step data. These participants had an average of 26.2 (SD = 6.7) days of step data out of the 30-day program. A small fraction of these days (40 / 8265, 0.5%) were generated by imputation using available data on steps per day the same week and same day of the week. The final model indicated that greater steps per day of retention was associated with higher academic status, less substantial change desired from program, higher step goal, greater frequency of engagement, greater number of friends, and team affiliation (Table S1).

| **Table S1.** Multiple variable regression analysis of person-level average daily steps before abandonment among the 315 participants who synced at least 4 days of step data | | | |
| --- | --- | --- | --- |
|  | **Least Squares Mean**  **± Standard Error** | **Mean Difference**  **(95% Confidence Interval)** | ***P*-value** |
|  |  |  |  |
| **Academic Status** |  |  | **.02** |
| Undergraduate student | 7138±311 | -834 (-1536, -133) | **.02** |
| Graduate Student | 6858±444 | -1115 (-2033, -198) | **.02** |
| Faculty/staff | 7973±352 | Reference |  |
| **Motive for joining** |  |  | **<.001** |
| Maintain current physical activity levels | 8202±307 | 1833 (968, 2700) | **<.001** |
| Increase physical activity levels | 7399±359 | 1031 (103, 1959) | **.03** |
| Start to become physically active | 6368±439 | Reference |  |
| **Goal** |  |  | **<.001** |
| 12,500 | 8708±552 | 2961 (1867, 4056) | **<.001** |
| 10,000 | 7515±318 | 1769 (1098, 2440) | **<.001** |
| 6,500 | 5746±278 | Reference |  |
| **App engagements per day before abandonment** |  |  | **<.001** |
| > 1.00 | 8219±287 | 1683 (811, 2556) | **<.001** |
| 0.50 ~ 1.00 | 7216±407 | 680 (-299, 1660) | .17 |
| <0.50 | 6535±436 | Reference |  |
| **Teammates made** |  |  | .48 |
| 9+ | 7459±289 | 528 (-484, 1539) | .31 |
| 5-8 | 7579±333 | 648 (-414, 1710) | .23 |
| 0-4 | 6931±516 | Reference |  |
| **Friends made** |  |  | **.01** |
| 4+ | 7853±414 | 1210 (354, 2066) | **.01** |
| 1-3 | 7474±336 | 831 (84, 1577) | **.03** |
| 0 | 6643±341 | Reference |  |
| Model adjusted for team affiliation, which had intra-cluster correlation coefficient (ICC) of .11.  Bolded highlights *P*<.05. | | | |
