# Supplementary material for: College Community–Based Physical Activity Support at a Public University During the COVID-19 Pandemic: Retrospective Longitudinal Analysis of Intra- Versus Interpersonal Components for Uptake and Outcome Association
Source: JMIR Mhealth Uhealth. 2025 Jun 16;13:e51707. doi: 10.2196/51707 (PMC12209730; doi:10.2196/51707)
Supplement: Multimedia Appendix 4 [file mhealth_v13i1e51707_app4.docx]

| **Table S2.** Demographics of Southern Connecticut State University as of October 15, 2020. | | | |
| --- | --- | --- | --- |
|  | **Undergraduates** | **Graduates** | **Faculty/staff** |
| **Total** | 7,440 | 1,891 | 1,724 |
| **Women** | 4,653 (62.5%) | 1,408 (74.5%) | 986 (57.2%) |
| **Age <25yr** | 6,361 (85.5%) | 834 (44.1%) | Not tracked |
| **Race/Ethnicity** |  |  |  |
| Nonresident alien | 72 (1.0%) | 24 (1.3%) | 4 (0.2%) |
| Hispanic/Latino | 1027 (13.8%) | 181 (9.6%) | 100 (5.8%) |
| American Indian or Alaskan Native | 12 (0.2%) | 5 (0.3%) | 2 (0.1%) |
| Asian | 253 (3.4%) | 41 (2.2%) | 100 (5.8%) |
| Black or African American | 1,374 (18.5%) | 202 (10.7%) | 228 (13.2%) |
| Native Hawaiian or other Pacific Islander | 4 (0.1%) | 0 (0.0%) | 0 (0.0%) |
| White | 3,795 (51.0%) | 1,243 (65.7%) | 1,249 (72.4%) |
| Two or more races | 356 (4.8%) | 57 (3.0%) | 12 (0.7%) |
| Unknown | 547 (7.4%) | 138 (7.3%) | 29 (1.7%) |
| Data are from the Integrated Postsecondary Education Data System by the National Center for Education Statistics [60]. | | | |
